# Coupled Transcription-Translation in Prokaryotes: An Old Couple With New Surprises

**DOI:** 10.3389/fmicb.2020.624830

**Published:** 2021-01-21

**Authors:** Mikel Irastortza-Olaziregi, Orna Amster-Choder

**Affiliations:** Department of Microbiology and Molecular Genetics, Faculty of Medicine, IMRIC, The Hebrew University of Jerusalem, Jerusalem, Israel

**Keywords:** coupled transcription-translation, uncoupled transcription-translation, subcellular organization of prokaryotes, translation-independent mRNA localization, local translation, expressome

## Abstract

Coupled transcription-translation (CTT) is a hallmark of prokaryotic gene expression. CTT occurs when ribosomes associate with and initiate translation of mRNAs whose transcription has not yet concluded, therefore forming “RNAP.mRNA.ribosome” complexes. CTT is a well-documented phenomenon that is involved in important gene regulation processes, such as attenuation and operon polarity. Despite the progress in our understanding of the cellular signals that coordinate CTT, certain aspects of its molecular architecture remain controversial. Additionally, new information on the spatial segregation between the transcriptional and the translational machineries in certain species, and on the capability of certain mRNAs to localize translation-independently, questions the unanimous occurrence of CTT. Furthermore, studies where transcription and translation were artificially uncoupled showed that transcription elongation can proceed in a translation-independent manner. Here, we review studies supporting the occurrence of CTT and findings questioning its extent, as well as discuss mechanisms that may explain both coupling and uncoupling, e.g., chromosome relocation and the involvement of cis- or trans-acting elements, such as small RNAs and RNA-binding proteins. These mechanisms impact RNA localization, stability, and translation. Understanding the two options by which genes can be expressed and their consequences should shed light on a new layer of control of bacterial transcripts fate.

## Coupled Transcription-Translation: A Hallmark Feature of Prokaryotic Gene Expression

**Figure 1:**
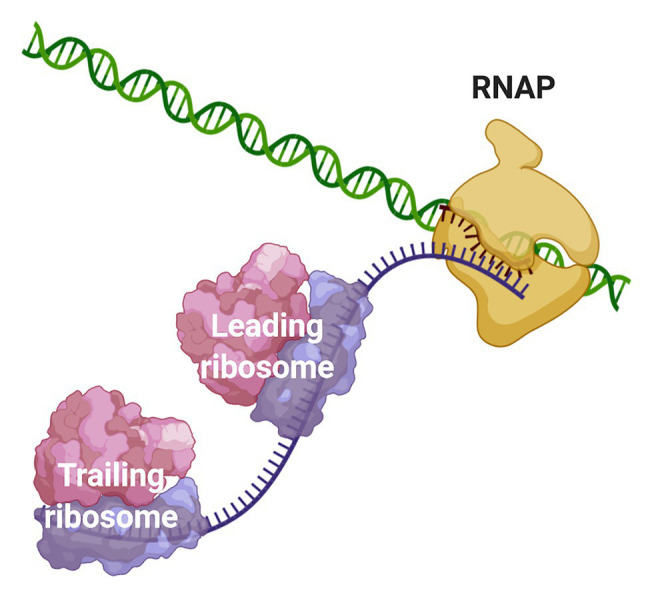
Coupling of transcription and translation in prokaryotes. When the nascent mRNA emerges from the RNAP, the transcript is bound by the leading ribosome forming a transcribing-translating complex. Additional ribosomes can associate with the nascent mRNA to form a convoy of trailing ribosomes on the transcript that is still bound to the transcription machinery. The leading ribosome can physically interact with the RNAP or the two machineries may be connected *via* the transcript.

Due to the scarcity of intracellular membrane-delimited compartmentalization, prokaryotic cells have historically been regarded as spatially unorganized. The lack of a nuclear membrane that physically separates the chromosomal DNA from the cytosolic environment led to the well-accepted notion that transcription and translation are spatiotemporally coupled in bacteria and archaea. Coupled transcription-translation (CTT) occurs when ribosomes bind and start to translate nascent mRNAs, whose transcription has not terminated yet, therefore forming an “RNAP·nascent mRNA·ribosome” complex (Figure 1).

The fact that transcription and translation could be coupled in prokaryotes was first proposed by Stent in the mid-1960s. He argued that due to the apparent inability to dissociate nascent transcripts from the chromosome *in vitro*, an active force exerted by translating ribosomes could be necessary to release the mRNAs from their templates. Indirectly, he implied that transcription and translation could be spatiotemporally coupled (Stent, 1964). Subsequent *in vitro* work from the Nirenberg lab demonstrated DNA·RNA·ribosome complexes (Byrne et al., 1964; Bladen et al., 1965). In the early 1970s, Miller et al. (1970) published an electron microscopy image, which showed ribosomes strongly associating with and translating nascent mRNA in a concatenated fashion forming polysomes. More recently, similar observations were reported in archaea (French et al., 2007), extending the occurrence of CTT to all prokaryotes. Miller’s micrographs have illustrated microbiology textbooks for decades and CTT is nowadays a widely accepted dogma of prokaryotic gene expression.

In this review, we will first highlight the biological significance of maintaining proper CTT and present the current understanding of how transcription and translation are coordinated under different growth conditions. We will then discuss the recent findings that shed light on the mechanistic and molecular details that mediate the physical coupling of these two processes. We will proceed by describing different models explaining where CTT takes place in the context of the prokaryotic cell. We will then introduce several types of evidence that challenge the CTT dogma and suggest that its occurrence may not be as general as currently assumed. Furthermore, we will extensively elaborate on molecular mechanisms that potentially promote the spatiotemporal uncoupling of transcription and translation. We will conclude by discussing open questions and principles emanating from this old but still exciting couple.

## Coordination of CTT

In optimal growth conditions, *Escherichia coli* ribosomes translate 14–17 amino acids per second (Young and Bremer, 1976; Proshkin et al., 2010; Zhu et al., 2016), meaning that they translocate about 42–51 nucleotides per second (nt/s) along the mRNA being translated. On the other hand, RNAP synthesizes mRNA at a rate of 42–49 nt/s (Proshkin et al., 2010; Iyer et al., 2018). Thus, mRNA transcription and translation rates are well-matched. Of note, translation and transcription rates vary across different growth conditions, but the rates of both processes remain coordinated (Vogel and Jensen, 1994b; Proshkin et al., 2010; Iyer et al., 2018), suggesting CTT coordination is important.

Indeed, a balanced CTT regime is crucial for the proper function of *E. coli* cells, and the uncoupling of transcription and translation can lead to multiple conflicts that compromise cell viability. Many such conflicts are related to the intimate link between CTT and Rho-mediated premature transcription termination (PTT). It was assumed for years that, in the absence of ribosomes engaged in CTT, Rho utilization (*rut*) sites in the nascent transcripts become exposed and are readily recognized by Rho, which translocates towards RNAP and causes the disassembly of the transcription elongation complex (TEC; Chalissery et al., 2011; Lawson et al., 2018). Recent evidence, on the other hand, supports a different mechanism where, in the absence of a physically coupled ribosome, transcription termination factor Rho associates with RNAP early after transcription initiation (Mooney et al., 2009a) *via* protein-protein interactions with NusA and NusG, rendering the TEC into a moribund pre-termination complex (PTC). This state favors Rho recognition of the *rut* sites in the nascent mRNA, which is followed by the closure of the Rho ring and disassembly of the TEC (Epshtein et al., 2010; Hao et al., 2020; Said et al., 2020). These molecular events lead to PTT and operon polarity (Richardson, 1991). Ribosomes and Rho compete for the same binding interface of NusG and, thus, a coupled ribosome prevents Rho-mediated PTT (Burmann et al., 2010). Coupled ribosomes can additionally antiterminate intrinsic terminators (Li et al., 2015). Moreover, leading ribosomes push stalled RNAPs forward and facilitate elongation over transcriptional roadblocks (Proshkin et al., 2010; Stevenson-Jones et al., 2020). Beyond gene expression, this is especially important for avoiding clashes between replisomes and backtracked RNAPs, which cause double-strand breaks and lead to genome instability (Dutta et al., 2011). Furthermore, cotranscriptional translation prevents reannealing of the nascent RNA to the template DNA strand, which can give way to dangerous R-loops (Gowrishankar and Harinarayanan, 2004), and also protects nascent transcripts from ribonucleolytic attack (Makarova et al., 1995; Deana and Belasco, 2005). In agreement with this, the stability of *lacZ* transcripts was greatly reduced when transcribed by T7 RNAP (Iost and Dreyfus, 1995), which transcribes about 230 nt/s (Golomb and Chamberlin, 1974) and cannot be closely followed by translating ribosomes. Tight CTT could be of critical importance for the expression of genes whose decay initiates before the transcription of the full mRNA is completed (Chen et al., 2015). For this population of transcripts, post-transcriptional translation appears to be unfeasible and ribosomes should closely follow RNAP before ribonucleases trigger the degradation of the nascent mRNA. Additionally, it is speculated that physically associated ribosomes can perform a pioneering round of translation for mRNA quality control, a well-characterized phenomenon in eukaryotes (Maquat et al., 2010). On the other hand, controlled uncoupling of transcription and translation can also be utilized for gene regulation. A classical example of this is the operon for tryptophan biosynthesis (Yanofsky, 1981), where due to low tryptophan concentrations the leader ribosome lags behind RNAP and allows the formation of an antitermination secondary structure that precludes the formation of the attenuator structure.

rRNA transcription (90 nt/s; Vogel and Jensen, 1994b) exceeds by far the translation elongation rates in *E. coli*, implying that, when transcribing mRNA, RNAP does not work at its maximum biosynthetic capacity, and that mechanisms for equalizing mRNA transcription and translation exist. In this regard, physical CTT offers an elegant model to explain the transcription-translation correlation under different growth conditions. According to this model, translation elongation rates of the leading ribosome, dictate transcription elongation rates (Proshkin et al., 2010). This model is supported by the finding that in strains harboring ribosomal mutations that slow translation, transcription elongation rates decreased accordingly, and that transcription-translation rates of different genes correlated with their rare codon content, highlighting the role of translation in dictating transcription elongation (Proshkin et al., 2010). In this model, the leading ribosome equalizes transcription-translation rates by physically pushing forward the RNAP, which otherwise tends to spontaneous backtracking and/or pauses (Proshkin et al., 2010; Stevenson-Jones et al., 2020), so that CTT remains coordinated and futile transcription is prevented. Of note, this mode of CTT coordination does not necessarily imply persistent RNAP·ribosome interactions or the formation of a stable complex, and it could rather be driven by occasional ribosome-to-RNA pushing contacts at sites where transcription slows down.

Besides CTT coordinated by physical RNAP·ribosome contacts, recent evidence argues in favor of additional mechanisms for CTT coordination. In contrast to common thought, chloramphenicol does not inhibit translation by reducing ribosome elongation rates, but rather by diminishing the population of ribosomes engaged in translation (Dai et al., 2016). In contrast, cells challenged with fusidic acid showed slower translation elongation rates, but strikingly, no reduction in transcription elongation rates (Zhu et al., 2019), suggesting that transcription elongation can be modulated independently to translation. Also, in cells subjected to nitrogen starvation, which is characterized by a slowdown in translation elongation rates, transcription elongation slowed down correspondingly and no hints of PTT were observed (Iyer et al., 2018). Importantly, translation inhibition by chloramphenicol did not decrease transcription elongation rates under these experimental conditions (Iyer et al., 2018), indicating that the RNAP elongates independently of the leading ribosome. Possibly, under conditions where translation slows down below a certain threshold, transcriptional cooperation among RNAPs (see section The CTT Dogma has Been Challenged: Towards Uncoupled Transcription-Translation?) might suffice to maintain transcriptional elongation in a ribosome-independent manner. Additional studies support a model where the occurrence of CTT is a stochastic event that does not depend on the physical contact of both machineries (see section The CTT Dogma has Been Challenged: Towards Uncoupled Transcription-Translation?; Li et al., 2015; Chen and Fredrick, 2018, 2020). Thus, it is reasonable to argue that, besides CTT coordinated by physical contacts between the leading ribosome and RNAP, additional mechanisms ensure the coordination of CTT.

Notably, the alarmone (p)ppGpp has recently emerged as an important player in CTT coordination. Upon amino acid starvation, the ribosome-associated RelA detects uncharged tRNAs at the A-site of the ribosomes and triggers the rapid synthesis of (p)ppGpp (Wendrich et al., 2002), inducing stringent response. Traditionally (p)ppGpp has been linked to transcriptional regulation, primarily by binding to the interface between β and ω subunit of the RNAP (Mechold et al., 2013; Zuo et al., 2013) and negatively regulating rRNA transcription, as well as a transcription of a myriad of coding sequences (Potrykus and Cashel, 2008; Sanchez-Vazquez et al., 2019). In agreement with this, in cells treated with fusidic acid that maintained normal transcription (p)ppGpp accumulation caused a slowdown of transcription elongation in an alarmone dose-dependent manner (Zhu et al., 2019). Alarmone-dependent slowdown of transcription elongation was also observed in cells subjected to amino acid starvation (Vogel et al., 1992; Vogel and Jensen, 1994a) and nitrogen limitation (Iyer et al., 2018).

Recent publications have expanded the (p)ppGpp targetome (Zhang et al., 2018; Wang et al., 2019), and additional alarmone-mediated layers of regulation have been discovered (reviewed in Hauryliuk et al., 2015). For instance (p)ppGpp competes with GTP for binding translation initiation factor 2 and elongation factor G (EF-G; Milon et al., 2006; Mitkevich et al., 2010), inhibiting their activity. Interestingly, under nitrogen starvation, ribosome stalling at glutamine codons diminishes translation elongation, and alarmone-defective strains show even slower translational elongation rates compared to wild-type cells (Li et al., 2018). Apparently, bacteria prevent generalized translation initiation, which could deplete pools of charged tRNAs and lead to incomplete translational rounds, and instead ensure that basal translation of housekeeping and stress-responsive genes remains active (Dai et al., 2016). This phenomenon has been proven essential for survival under oxidative stress, which is also characterized by a dramatic slowdown of translation elongation rates (Zhu and Dai 2019, 2020). It was recently reported that (p)ppGpp-dependent translational selectivity depends on structural motifs formed by the mRNAs (Vinogradova et al., 2020). Thus, upon environmental challenges that reduce translation elongation rates, cells achieve CTT coordination by slowing down RNAP elongation and by prioritizing translation of a subset of essential genes that avoid alarmone-mediated translation inhibition. Highlighting the importance of this regulation is the finding that disrupting (p)ppGpp-mediated CTT coordination sensitizes cells to nitrogen starvation (Iyer et al., 2018).

Alarmones also coordinate transcription and translation less straightforwardly. It has recently been shown that (p)ppGpp binds inosine-guanosine kinase, which prevents the excessive accumulation of purine nucleotides and leads to pRpp synthesis, a precursor molecule for pyrimidine nucleotides, tryptophan, and histidine (Wang et al., 2020a). Thus, alarmones further coordinate CTT indirectly, by balancing nucleotide stocks required for transcription and ensuring tryptophan and histidine availability to maintain basal translation. Interestingly, the operons for the biosynthesis of these amino acids are regulated by transcriptional attenuation (Yanofsky, 1981), which links their regulation to sensing the CTT status.

**Figure 2:**
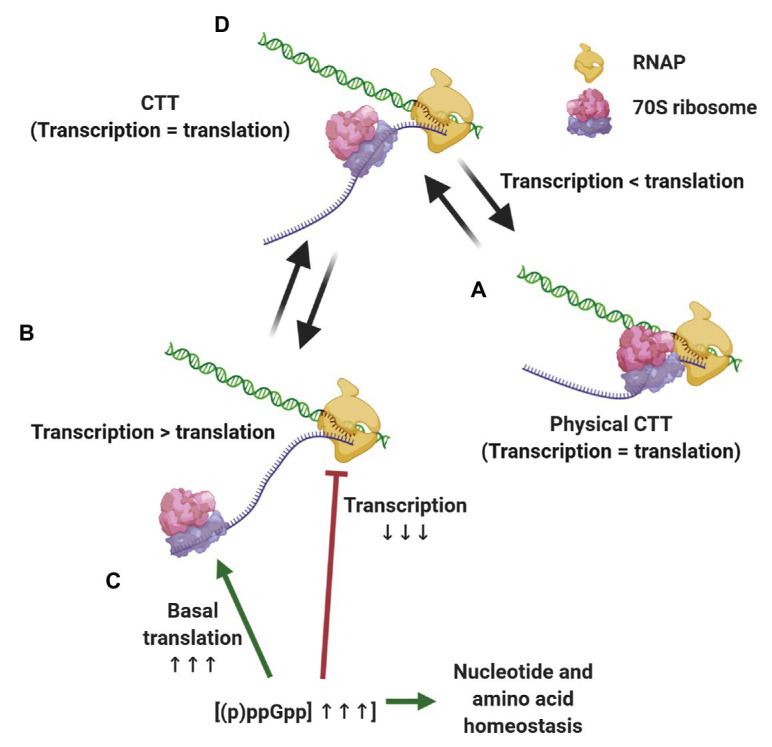
Strategies for maintaining coupled transcription-translation (CTT) coordination. Under conditions that slow down transcription elongation rates below translational rate, such as carbon limitation and RNAP backtracking **(A)**, the leading ribosome catches up with and physically pushes the transcription elongation complex (TEC) forward, equalizing transcription and translation rates. When translation elongation lags behind transcription rate, e.g., upon nitrogen limitation and oxidative stress **(B)** (p)ppGpp concentrations increase, slowing down transcription elongation, promoting basal translation and balancing nucleotide and amino acid stocks **(C)**, thus equalizing transcription and translation elongation rates **(D)**.

The evidence presented above supports a scenario where CTT coordination is preserved by different, although complementary means, depending on the growth conditions (Figure 2). When transcription slows down below translation elongation rates, due to spontaneous backtracking or pausing, the leading ribosome physically pushes the RNAP forward in a way that translation rates dictate equal transcription elongation rates (Proshkin et al., 2010; Stevenson-Jones et al., 2020). Under conditions where ribosome elongation lags behind transcription, such as nitrogen limitation or oxidative stress, multilayered regulation *via* (p)ppGpp ensures that RNAP elongation rates decrease accordingly and maintains CTT coordination without the need for physical contacts of the transcription and translation machineries. Considering the ongoing discovery of (p)ppGpp-regulated processes and the tight relationship of alarmones with the metabolic status of the cell, we expect that additional pathways ensuring CTT coordination *via* (p)ppGpp will be discovered. These complementary mechanisms, i.e., physical contacts between RNAP and the leading ribosome and alarmone-mediated coordination, account for the observed correlation between transcription and translation elongation rates under different growth conditions.

## The Molecular Architecture of CTT

How do the transcriptional and translational machineries intimately interact? Several recent publications have shed light on the molecular interactions that mediate the physical coupling of transcription and translation in *E. coli*, although their conclusions were not always in agreement (reviewed by Conn et al., 2019). CTT was initially proposed to be mediated by NusG. Specifically, nuclear magnetic resonance (NMR) experiments showed that the N-terminal domain (NTD) and C-terminal domain (CTD) of NusG interact with RNAP and NusE, which doubles as S10 protein in the ribosome (Mooney et al., 2009b; Burmann et al., 2010), respectively, thus, bridging the transcriptional and translational machineries. As Rho and the leading ribosome compete for NusG binding, this mode of coupling also explains why TECs engaged in CTT avoid Rho-mediated termination (Burmann et al., 2010). CTT of a subset of horizontally acquired operons was shown to be bridged by the NusG homolog RfaH in a similar fashion (Burmann et al., 2012; Zuber et al., 2019). The NusG-dependent CTT model was further supported by *in vivo* and *in vitro* experiments evidencing that NusG simultaneously interacts with the ribosome and RNAP (Saxena et al., 2018). A cryo-electron microscopy (cryo-EM) structure of a TEC·NusG complex did not show any defined density for the NusG CTD, suggesting that NusG mediates a flexible RNAP-ribosome association (Kang et al., 2018b). This was confirmed by another cryo-EM structure of a ribosome-bound NusG, that showed a defined density for the CTD but not for the NTD (Washburn et al., 2020). This latter publication also reported NMR results supporting the association of an RNAP·NusG complex with S10.

In parallel, a series of publications posited a model where transcription and translation are coupled independent of NusG, *via* direct RNAP·ribosome interactions. Chemical cross-linking experiments demonstrated multiple NusG-independent interactions between RNAP and both 30S and 50S ribosome subunits (Fan et al., 2017), and an RNAP·30S subunit cryo-EM structure (Demo et al., 2017) recapitulated several of the interactions detected by cross-linking. Simultaneously, a cryo-EM structure of a transcribing-translating RNAP·ribosome complex, named expressome, showed that direct interactions between both machineries can mediate the coupling (Kohler et al., 2017). Different from the previous works described above, this structure was produced by colliding a translating ribosome against a transcriptionally stalled RNAP. In this structure, the exit site of RNAP and the mRNA entry site of the ribosome were closely placed, which suggests continuous protection of the nascent mRNA from transcription to translation. This protection also excludes Rho from accessing nascent transcripts and from terminating transcription. Importantly, the NusG binding partners in the CTT complex, i.e., the β'-subunit of RNAP and the S10 protein of the ribosome, were located on opposite sites of the complex and the NusG linker was too short to bridge such distance. Thus, the collided expressome was not compatible with NusG-mediated CTT (Kohler et al., 2017).

How can the divergent views of NusG-dependent and -independent CTT, both emerging from structural studies, be reconciled? Two simultaneous studies presenting cryo-EM structures suggest that these views can co-exist. The first study by the Weixlbaumer lab describes the structures of transcribing-translating complexes assembled on mRNA scaffolds that allow different distances between the RNAP active site and the ribosomal P-site (Webster et al., 2020). One structure showed, for the first time, simultaneous binding of the NTD and CTD of NusG to RNAP and the leading ribosome, correspondingly. In this structure NusG forms a bridge between the ribosome and RNAP, thus stabilizing the interaction interface between the two machineries. Increasing the length of the intervening mRNA resulted in a similar structure, i.e., a NusG-coupled expressome. However, shortening of the intervening mRNA resulted in a structure in which RNAP is located closer to the ribosome entry tunnel. In this collided expressome, RNAP could still bind NusG, but the latter was not able to bridge the distance to S10. Hence, as shown previously (Kohler et al., 2017), the collided expressome is not compatible with NusG-mediated CTT. Of note, a structure nearly identical to this collided expressome was obtained when assembling expressomes on short scaffolds in the absence of NusG (Kohler et al., 2017). The conclusions that emerge from these structures are that coupling *via* NusG restrains RNAP motions, that the length of the intervening mRNA determines whether NusG can be involved, that expressome formation is strictly mRNA-dependent, although RNAP·30S complexes were previously observed (Demo et al., 2017), and that both the NusG-bridged and the collided expressomes are compatible with translation factor binding (Webster et al., 2020).

The second study, published by the Ebright lab, in addition to confirming many of the results presented by Weixlbaumer and coworkers and showing that collided expressomes are incompatible with ribosome·NusG binding, provides insights into the participation of NusA in CTT (Wang et al., 2020b). The researchers’ present high-resolution structures for expressomes that involve both NusG and NusA in CTT. In these structures, which accommodated spacer mRNAs of different lengths, NusA promoted expressome assembly by acting as a “coupling pantograph” between RNAP and the S2/S5 protein of the leading ribosome.

Based on molecular modeling, Ebright and co-workers point out several features of the collided expressome that question its capability to promote proper gene expression (Wang et al., 2020b). First, the collided expressome is sterically incompatible with NusA binding (Guo et al., 2018), with the formation of Q-dependent antitermination complexes (Shi et al., 2019; Yin et al., 2019) and with the formation of pause and termination RNA hairpins (Kang et al., 2018a; Roberts, 2019). Regarding translation, the collided expressome is not compatible with the swiveling of the 30S subunit head that takes place during ribosome translocation (Schuwirth et al., 2005; Ratje et al., 2010; Guo and Noller, 2012). Furthermore, collided expressomes lack densities corresponding to the RNAP ω subunit, which assists in TEC assembly and, by binding (p)ppGpp, mediates CTT coordination during stringent response (see section Coordination of CTT; Kurkela et al., 2020). Thus, it is highly unlikely that collided expressomes are responsible for general CTT and, instead, they could be specialized complexes in charge of CTT under specific conditions, or even anomalous complexes resulting from RNAP-ribosome clashes (Wang et al., 2020b). Supporting the latter, RNAP from a collided expressome was shown to reinitiate transcription elongation *in vitro* and detach from the ribosome that is purposely stalled (Stevenson-Jones et al., 2020), suggesting that it is not a stable complex with significant biological functions.

**Figure 3:**
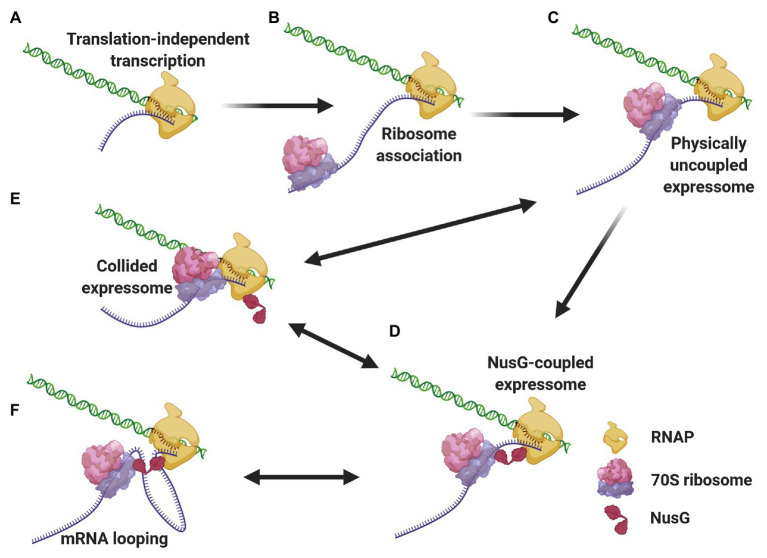
Different expressome configurations suggested to engage in gene expression. Transcription initiates over 5'-proximal coding sequences, eventually forming the TEC **(A)**. After considerable elongation, the leading ribosome associates with the nascent transcript and initiates translation **(B)**. RNAP pauses, allowing the leading ribosome to catch up with the TEC, forming a physically-uncoupled expressome **(C)**. The proximity of the RNAP and the leading ribosome allows for NusG association with both machineries, forming a NusG-bridged coupled expressome **(D)**. Transcriptional roadblocks can reduce the length of the connecting mRNA, giving way to a collided expressome, either with or without NusG **(E)**. Conversely, translational slowdown lengthens the connecting mRNA, which can loop out the complex **(F)**. The two latter events are reversible, giving way to a rearrangement of the coupled expressome.

We propose a mechanistic model (Figure 3) where different expressomes could come into play sequentially during different stages of *E. coli* CTT. Translation initiation is a relatively lengthy process (median time of 15–30 s; Siwiak and Zielenkiewicz, 2013; Shaham and Tuller, 2018) compared to transcription elongation (49 nt/s; Iyer et al., 2018), so according to our model, RNAP can elongate several dozens, if not hundreds of nucleotides beyond the start codon translation-independently before the leader ribosome initiates elongating. Once the leading ribosome begins elongating, the two machineries are remotely connected by the nascent mRNA as an uncoupled expressome. The two machineries could remain kinetically coupled and the uncoupled expressome could conduct gene expression under conditions where the non-physical coupling is coordinated *via* (p)ppGpp (see section Coordination of CTT). In other cases, this kinetic coupling precedes the formation of a coupled expressome. Specifically, RNAP tends to pause within the first 100 nt after the start codon (Mooney et al., 2009a; Larson et al., 2014), which can allow the leading ribosome to catch up with the TEC. Several types of data indicate that ribosome translocation along the nascent mRNA to the proximity of the TEC may be a prerequisite for the formation of a physically coupled expressome. Firstly, NusG association with TECs, which occurs only after substantial transcription (Mooney et al., 2009a), is facilitated by cotranscriptional translation (Washburn et al., 2020). Additionally, the intranucleoidal ribosome concentration (2–8 μM; Bakshi et al., 2012; Sanamrad et al., 2014) is substantially lower than the NusG·ribosome dissociation constant (50 μM; Burmann et al., 2010), so the NusG·ribosome association is likely favored by the translocation of the leading ribosome towards the TEC along the nascent mRNA. Thus, the arrangement of the uncoupled expressome promotes interactions of NusG with both machineries and favors the formation of a coupled expressome. This complex could be further stabilized by NusA (Wang et al., 2020b), which associates with elongating TECs early after transcription initiation (Mooney et al., 2009a).

The coupled expressome may proceed with CTT. Yet, transcriptional and translational elongation rates can respond to independent signals, resulting in varying lengths of the nascent mRNA connecting the RNAP and the leading ribosome (Conn et al., 2019). For instance, transcriptional pauses or backtracking reduce the distance between the leading ribosome and RNAP, which can give way to the formation of a collided expressome (Kohler et al., 2017; Wang et al., 2020b; Webster et al., 2020). As the leading ribosome pushes the stalled RNAP forward (Stevenson-Jones et al., 2020), the coupled expressome can be reconfigured. Such “collision-and-reconfiguration” events can also occur in the case of uncoupled expressomes. On the other hand, translational roadblocks will increase the distance between the leading ribosome and RNAP. As the mRNA connecting the two machineries in the coupled expressome is exposed to the solvent (Wang et al., 2020b; Webster et al., 2020), the longer nascent mRNA can be accommodated by looping out from the coupled expressome (Conn et al., 2019). Decreased transcription elongation and/or increased translation elongation reduce the length of the intervening mRNA and loop the protruding mRNA back into the complex.

The in-cell architectures of *Mycoplasma pneumoniae* expressomes published recently show similarities to the *E. coli* expressomes, as well as differences (O’Reilly et al., 2020). On one hand, structures obtained from pseudouridimycin-halted *M. pneumoniae* TECs, which likely represent collided expressomes, showed direct interactions between RNAP and the leading ribosome, similar to *E. coli* collided expressomes. On the other hand, *M. pneumoniae* elongating expressomes showed neither direct nor NusG-mediated interactions. Instead, the coupling between RNAP and the leading ribosome was mediated solely by NusA (O’Reilly et al., 2020). Thus, although direct RNAP·ribosome interactions, which characterize collided expressomes, seem a conserved phenomenon, the molecular actors and interactions that drive factor-mediated RNAP-ribosome coupling differ in evolutionarily unrelated species.

It should be emphasized that all *E. coli* expressome structures discussed in this section were obtained *in vitro*, and that their existence *in vivo* remains to be investigated. Also, the questions of whether RNAP and the lead ribosome are physically coupled on the nascent mRNA *in vivo* and whether there is a mechanism to ensure or promote this coupling remain open.

## The Cell Biology of CTT

Where in the bacterial cell does CTT occur? The subcellular organization of the transcriptional and translational machineries offers clues to answer this question. RNAP spends most of its lifetime bound to DNA, either engaged in transcription or non-specifically searching promoters (Endesfelder et al., 2013; Stracy et al., 2015; Ladouceur et al., 2020), and very few RNAP molecules are observed outside the nucleoid region (Bakshi et al., 2012). In rich media, RNAPs form nucleolus-like clusters engaged in rRNA transcription, but cluster assembly is independent of ongoing transcription (Jin et al., 2013; Gaal et al., 2016; Weng et al., 2019). Recently, it has been shown that RNAPs nucleate and form biomolecular condensates by liquid-liquid phase separation (LLPS; Ladouceur et al., 2020).

Ribosome localization, on the other hand, differs considerably among species. In organisms with a high nucleocytoplasmic (NC) ratio (Gray et al., 2019), such as *Caulobacter crescentus*, the ribosomes, and the nucleoid are homogeneously mixed (Bowman et al., 2010; Montero Llopis et al., 2010; Bayas et al., 2018). This facilitates the encounter of the transcriptional and translational machineries, and the occurrence of CTT can be envisaged fairly intuitively in these organisms. Yet, in many other species with low NC ratio, including *E. coli* (Hobot et al., 1985; Azam et al., 2000; Valencia-Burton et al., 2007; Wang et al., 2011; Bakshi et al., 2012; Chai et al., 2014; Cougot et al., 2014; Mohapatra and Weisshaar, 2018; Zhu et al., 2020), *Bacillus subtilis* (Lewis et al., 2000; Mascarenhas et al., 2001), *Bdellovibrio* (Borgnia et al., 2008), and *Pseudomonas putida* (Kim et al., 2019a), ribosomes and nucleoids are strongly segregated. The nucleoid-excluded localization of *E. coli* factors engaged in translation, such as tRNAs (Plochowietz et al., 2016; Volkov et al., 2018) and translation EFs (Chai et al., 2014; Mohapatra et al., 2017; Mustafi and Weisshaar, 2018), also supports that in these species bulk translation takes place in spatial separation from the genetic material and the transcriptional machinery. Hence, in these species, the transcriptional and translational machineries rarely encounter each other and the occurrence of CTT is less intuitive.

Several biophysical forces cause the subcellular nucleoid-vs-ribosome segregation. By avoiding extensive contacts with the inner membrane, the DNA polymer maximizes its number of available conformational states, i.e., the conformational entropy, and by segregating from the nucleoid, the ribosomes optimize their freedom for motion and the translational entropy (Mondal et al., 2011; Bakshi et al., 2014). This segregation is further accentuated by volume exclusion forces mutually exerted by the nucleoid polymer against the bulky polysomes (Mondal et al., 2011; Bakshi et al., 2014; Castellana and Wingreen, 2016). Lastly, electrostatic repulsion forces between negatively charged nucleic acids, in this case, chromosomal DNA and RNA-rich ribosomes (Joyeux, 2016), and phase separation effects (Joyeux, 2018) could also account for the observed antilocalization of nucleoids and ribosomes.

**Figure 4:**
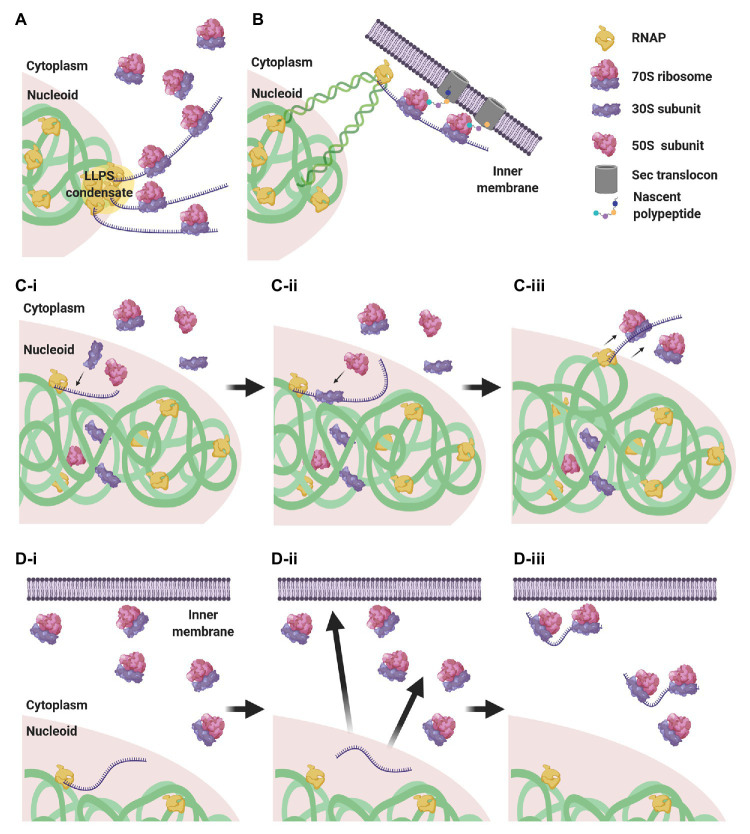
CTT scenarios in low nucleocytoplasmic (NC) ratio species. **(A)** RNAP clusters form condensates by liquid-liquid phase separation (LLPS) that are expelled to the nucleoid periphery, where they encounter ribosomes that engage in CTT. **(B)** *Via* transertion, gene loci encoding membrane proteins emerge from the nucleoid to the inner membrane, where CTT readily occurs. **(C)** Intranucleoidal translation initiation. Free ribosomal subunits penetrate the nucleoid and a 30S subunit associates with the SD sequence of the nascent transcript **(C-i)**. A 50S subunit associates with the pre-initiation complex, forming a 70S ribosome **(C-ii)**. The RNAP·mRNA·ribosome complex is expelled to the nucleoid periphery, where CTT proceeds **(C-iii)**. **(D)** Uncoupled transcription-translation (UTT). Transcription takes place within the nucleoid translation-independently **(D-i)**, and ribosome-free transcripts navigate the cell to their corresponding destination in the cytoplasm or in the membrane **(D-ii)**, where they are locally translated **(D-iii)**.

Considering this subcellular segregation of RNAPs and ribosomes, how does CTT occur in these organisms? One possibility is that CTT occurs at the surface of the nucleoid, where both RNAPs and ribosomes may encounter each other (Figure 4A). Indeed, it was shown that highly transcribed gene loci migrate to the nucleoid periphery (Stracy et al., 2015; Weng et al., 2019; Yang et al., 2019). The association of several RNAPs into biomolecular condensates at highly transcribed loci (Ladouceur et al., 2020) could cause the exclusion of these condensates from the nucleoid surface by the biophysical forces described above. Supporting this hypothesis, the cotranscriptional association of bulky ribosomes amplifies loci migration to the nucleoid periphery (Yang et al., 2019).

Gene loci encoding membrane proteins are thought to be expressed by coupled transcription-translation-membrane insertion, a mechanism known as transertion (Woldringh, 2002). This implies that certain gene loci migrate from the nucleoid mesh to the vicinity of the inner membrane and become exposed to ribosome-rich regions, where CTT could readily occur (Figure 4B). Although the occurrence of transertion still awaits direct experimental validation (Roggiani and Goulian, 2015), the notion is supported by the demonstration that a small population of ribosomes and RNAPs resides in the membrane vicinity (Herskovits and Bibi, 2000; Bakshi et al., 2012), and by the visualization of induction-dependent membrane relocation of several gene loci coding for membrane proteins (Libby et al., 2012; Kannaiah et al., 2019; Yang et al., 2019). Thus, localizing TECs to ribosome-rich regions *via* transertion may very well promote the encountering of RNAPs and ribosomes and facilitate CTT.

Alternatively, it was shown in *E. coli* that, although 70S ribosomes are segregated from the nucleoid, free 30S and 50S subunits can penetrate the nucleoid mesh (Sanamrad et al., 2014; Mohapatra and Weisshaar, 2018; Zhu et al., 2020). This implies that canonical translation initiation, which is conducted by a free 30S subunit that recognizes the Shine-Dalgarno (SD) sequence of the nascent transcript, could initiate within the nucleoid in a cotranscriptional manner. Upon assembly of the 70S monosome, the RNAP·nascent mRNA·ribosome complex would be pushed to the surface of the nucleoid by the biophysical forces mentioned above, where additional ribosomes could engage in CTT (Figure 4C).

An additional possibility is that transcription and translation are not compulsorily coupled. In this scenario, transcription takes place within the nucleoid, in spatiotemporal separation from translation, and the mRNAs then navigate the cytoplasm to their final destination where they are locally translated (Kannaiah and Amster-Choder, 2014; Irastortza-Olaziregi and Amster-Choder, 2020; Figure 4D). We will elaborate on this scenario in section The CTT Dogma has Been Challenged: Towards Uncoupled Transcription-Translation?

## The CTT Dogma has Been Challenged: Towards Uncoupled Transcription-Translation?

Coupled transcription-translation is widely accepted by the microbiology community and supported by extensive work. Yet, most knowledge regarding CTT emanates either from *in vitro* studies or from experiments conducted with only a handful of genes. Importantly, the subcellular segregation of the transcriptional and translational machineries observed in some species raises the possibility that these processes could take place in spatiotemporal separation (see section The Cell Biology of CTT). Indeed, several lines of evidence have recently challenged the classical view that transcription is inherently coupled to translation, and the global occurrence of CTT has been questioned.

The Fredrick lab developed a hammerhead ribozyme-based reporter system that enables measuring and comparing protein synthesis carried out by limited vs. unlimited number of translation rounds (Chen and Fredrick, 2018). They applied this system to study the translation of six adjacent gene-pairs that are cotranscribed in the same operon. In a tight CTT scenario, limiting translation rounds should bring the relative protein amounts for each gene-pair close to 1:1, as protein synthesis of the two co-transcribed genes would presumably be carried out by a leading ribosome physically coupled to RNAP. However, for five out of six gene-pairs, they observed that the relative protein synthesis derived from limited translation rounds of the two genes was not close to 1:1. Rather, the ratio was similar to that measured for unlimited translation rounds. This indicates that the first translational rounds occur independently of transcription, i.e., they are not carried out by a ribosome that is physically bound to RNAP. Importantly, when these experiments were repeated with an RNAP mutant showing reduced transcription elongation rate, which, presumably, facilitates the physical coupling of the leading ribosome with RNAP (see section The Molecular Architecture of CTT), protein production after limited translation rounds was closer to 1:1 for one of the tested gene-pairs (Chen and Fredrick, 2018). These results support a model where the physical coupling of transcription and translation is a stochastic event, which depends on the rates of transcription and translation elongation. These observations indicate that physical association between the leading ribosomes and RNAPs, as well as coordinated elongation by the two machineries, is significantly less common than currently assumed, implying that RNAP often transcribes without a linked ribosome (Chen and Fredrick, 2020). Supporting this notion, pseudouridimycin, which stalls TECs, notably increased the percentage of ribosomes that are physically coupled to RNAP in *M. pneumoniae* (O’Reilly et al., 2020). This indicates that, in the absence of transcription-halting antibiotics, the majority of those ribosomes are engaged in CTT, but that they do not translate in physical association to RNAP.

Similar conclusions emanated from studying the relationship between termination efficiency (TE) at intrinsic terminators and their intragenic position (Li et al., 2015). TE of terminators located in the first 100 nt of ORFs was close to 100%, and it gradually decreased as the distance from the start codon increased. This position-dependent loss of TE was explained by the fact that ribosomes follow and catch up RNAPs closely enough to prevent the formation of terminators (Li et al., 2015). Indirectly, the dependence of TE on the distance from the start codon implies that transcription of the 5'-proximal coding sequences occurs translation-independently (see section The Molecular Architecture of CTT).

Whereas these studies argue against the idea of physical CTT, they are not incompatible with the existence of RNAP-ribosome complexes remotely connected by nascent mRNAs. Yet, further evidence supports the idea that transcription and translation in some cases are completely uncoupled. It is accepted that translation increases mRNA stability by ribosome shielding against ribonucleolytic attack (Iost and Dreyfus, 1995; Makarova et al., 1995; Deana and Belasco, 2005). A reassessment of RNA decay patterns unraveled that, contrary to the widespread idea that RNA decay is exponential, two-thirds of the analyzed transcripts followed a biphasic degradation pattern, with a very steep decay at short post-transcription times followed by an exponential decay at longer times (Deneke et al., 2013). These results suggest that most transcripts spend a minor fraction of their lifetime in a ribosome-free form, where they are highly vulnerable to ribonucleases until ribosomes engage in translation and slowdown mRNA decay. At the same time, similar to what happens in eukaryotic cells, other RNA-binding proteins could be responsible for the protection of the transcripts till they are translated. Whatever the reason is, these results imply that transcription is not tightly coupled to translation for most genes (Deneke et al., 2013).

Furthermore, previous work from our lab and others showed that transcripts can localize to sites overlapping with the localization of their encoded proteins in the cytoplasm, the membrane, or the poles of *E. coli* cells (Nevo-Dinur et al., 2011; Moffitt et al., 2016). Very importantly, these localization patterns were preserved when the translation of the tracked transcripts was inhibited by antibiotics, translational roadblocks, or mutations (Nevo-Dinur et al., 2011). Similarly, a transcript encoding a short membrane protein localized to the membrane even when the SD sequence of the mRNA was deleted (Steinberg et al., 2020). Likewise, translation-independent RNA localization has been observed in cyanobacteria, where transcripts encoding photosystem components localized to thylakoid membranes in the presence of puromycin concentrations that inhibit translation and detach ribosomes from mRNAs (Mahbub et al., 2020). These observations imply that bacterial mRNAs can skip tight CTT, navigate the cytosol as ribosome-free transcripts, and undergo local translation once they reach their corresponding destination (Nevo-Dinur et al., 2011; Kannaiah and Amster-Choder, 2014; Irastortza-Olaziregi and Amster-Choder, 2020). Another recent publication from our lab, which reports the distribution of *E. coli* RNAs between the membrane, cytoplasm, and poles by combining cell fractionation with deep-sequencing, showed that a significant fraction of the *E. coli* transcriptome localizes in a translation-independent manner and challenged the idea that CTT is a general mechanism for gene expression (Kannaiah et al., 2019). Collectively, this evidence supports the notion that CTT is not as predominant as currently assumed, and that spatiotemporally uncoupled transcription-translation (UTT) could be responsible for substantial gene expression.

UTT implies that processive transcription can occur independently of ribosomes that are coupled to RNAP by physical interaction, *via* protein factors or through mRNAs. For *E. coli* RNAPs, translation-independent transcription has been attributed only to TECs engaged in rRNA transcription, which are modified by the antitermination complex and show fast transcription elongation rates that outpace Rho and avoid termination (Squires and Zaporojets, 2000; Paul et al., 2004). For mRNA-transcribing TECs, the dogma that prevailed was that a coupled ribosome is required for transcription processivity (see section Coordination of CTT). Then again, early evidence already indicated that transcription can be tuned independently of translation. Although mRNA transcription-translation rates change and equalize each other at different growth rates, rRNA transcription rates also vary according to growth rates (Vogel and Jensen, 1994b), suggesting that ribosome-independent mechanisms exist in bacteria for determining transcription elongation rates. As discussed in section Coordination of CTT, the application of sublethal concentrations of fusidic acid, which slows down ribosome translocation, did not affect RNAP elongation rates (Zhu et al., 2019). Besides, when ribosomes stalled at proline-rich sequences of *E. coli* cells deleted for EF-P (Elgamal et al., 2016), or when (p)ppGpp-mediated CTT coordination was disrupted under nitrogen starvation (Iyer et al., 2018), RNAP was still able to elongate. In further agreement with ribosome-independent transcription, when backtracked TECs are pushed and reactivated by the leading ribosome, transcription elongation restarts even when translation elongation is inhibited (Stevenson-Jones et al., 2020). Likewise, in *M. pneumoniae* subjected to chloramphenicol treatment, the RNAP·ribosome association is lost (O’Reilly et al., 2020), which reinforces the idea that RNAP can detach from the expressome and transcription elongation proceeds independently to translation.

How can the transcription processivity of TECs that are engaged in mRNA transcription be maintained in the absence of a coupled ribosome? The trafficking of transcription elongation and termination factors can offer a partial explanation for this. NusG recruitment to *E. coli* TECs occurs after substantial transcription and is assisted by a translationally coupled ribosome (see section The Molecular Architecture of CTT). Although Rho is recruited to TECs early after transcription initiation (Mooney et al., 2009a), it still requires an RNAP-associated NusG for inducing the PTC before triggering termination (Epshtein et al., 2010; Hao et al., 2020; Said et al., 2020). Thus, translation-independent TECs could show less affinity for NusG and, consequently, may be less prone to Rho-mediated termination.

Cooperation among RNAPs (reviewed by Le and Wang, 2018) could further facilitate the processivity of ribosome-independent TECs. For instance, similar to a leading ribosome that pushes a backtracked RNAP forward (see section Coordination of CTT), trailing RNAPs facilitate transcription of the leading RNAP over DNA roadblocks and rescue backtracked TECs by physically pushing the preceding RNAP forward (Epshtein et al., 2003; Epshtein and Nudler, 2003). Effects related to DNA supercoiling offer alternative explanations for this transcriptional cooperation. A mathematical model predicts that the torque created by transcription elongation over the DNA double helix pushes and pulls TECs located in close proximity forward without the mediation of any physical contact among each other (Heberling et al., 2016). Recently, a publication from the Jacobs-Wagner lab evidenced that, as long as gene promoters remain induced and multiple RNAPs initiate transcription of the *lacZ* gene, elongation rates of ongoing transcription are maintained by the mutual cancelation of positive and negative DNA supercoiling upstream and downstream the TEC convoy (Kim et al., 2019b). Regardless of the actual underlying mechanism, we suggest that the cooperation between RNAPs could suffice for maintaining the transcription processivity required for UTT.

*In vitro* studies showed that the *B. subtilis* RNAP transcribes much faster than the *E. coli* RNAP (Artsimovitch et al., 2000) and, notably, a recent publication demonstrated that transcription elongation in *B. subtilis* outpaces ribosome translocation over nascent transcripts (Johnson et al., 2020). Although, as reported for *E. coli*, leading ribosomes could still physically push and rescue stalled or paused *B. subtilis* TECs (see section Coordination of CTT), this “runaway” transcription creates extensive distance between RNAP and the leading ribosome, supporting the idea that transcription and translation are mostly uncoupled in *B. subtilis*. Accordingly, transcription terminators in *B. subtilis* are located only a few nucleotides downstream of stop codons (Johnson et al., 2020), which would otherwise be masked by a ribosome physically associated with RNAP (Li et al., 2015). A bioinformatic exploration using this short distance between stop codons and intrinsic terminators as a proxy for runaway transcription suggested that this mode of gene expression could be a fairly widespread phenomenon in bacteria (Johnson et al., 2020), once again arguing against the universality of CTT (Wang and Artsimovitch, 2020).

## Mechanisms Potentially Enabling UTT

Considering the evidence presented in section The CTT Dogma has Been Challenged: Towards Uncoupled Transcription-Translation, it is plausible that CTT may not be as universal as has been assumed for many years. In other words, transcription may occur translation-independently, and mRNAs could be transcribed and translated in spatiotemporal separation. Yet, for UTT to take place, bacteria would need to face three major challenges: (1) Prevent association between the leading ribosome and the nascent mRNA, once the SD sequence emerges from RNAP. (2) Protect the ribosome-free transcript from ribonucleolytic decay in the cytoplasm. (3) Carry out translation-uncoupled transcription without disruption by intrinsic and Rho-dependent terminators.

Dynamics of transcription elongation over 5'-proximal coding sequences are of special interest for UTT. In *E. coli*, RNAP shows pronounced transcriptional pausing at sites overlapping with SD sequences and start codons (Larson et al., 2014), thus opening a temporal window for regulation over the naked 5'-UTRs by RNA-Binding proteins (RBPs) or RNA folding events prior to ribosome association with the transcript. Furthermore, transcription of 5'-proximal coding sequences is anyhow expected to occur translation-independently (see section The Molecular Architecture of CTT), so why cannot this type of regulation continue to be exerted over the downstream ribosome-naked transcript before translation begins?

**Figure 5:**
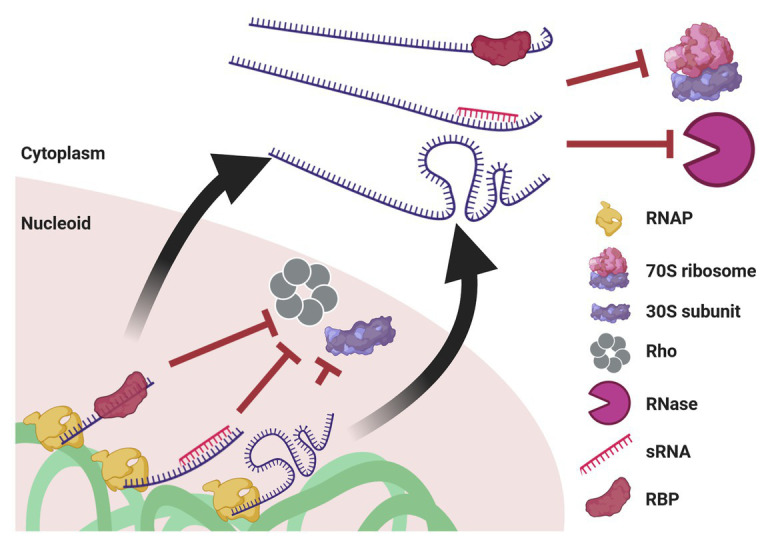
Mechanisms potentially enabling UTT. Cotranscriptional events, such as association with an RBP or with an sRNA, as well as riboswitch formation, prevent transcription termination by Rho and association with the leading ribosome. When ribosome-free transcripts are released to the cytoplasm, these transcript-protecting events counteract the activity of ribonucleases. Of note, although drawn linearly, transcripts supposedly acquire complex secondary and tertiary structures that confer further protection. This protection also prevents mRNA translation until they reach their final destination.

Below, we describe several molecular factors and mechanisms that, when acting in cooperation, potentially promote UTT in a way that the three challenges mentioned above would be satisfactorily resolved (Figure 5). Evidence supporting the capacity of these factors and mechanisms to act cotranscriptionally on nascent mRNAs is presented. Furthermore, formation of biomolecular condensates by LLPS and their putative implication in promoting UTT are briefly discussed.

### RNA-Binding Proteins

RBPs have gained major interest as a consequence of their capability to regulate transcript fate by affecting mRNA translation and stability (Holmqvist and Vogel, 2018; Richards and Belasco, 2019), and emerging evidence suggest they can promote UTT. For instance, *Synechocystis* RBPs Rbp2 and Rbp3 are required for the translation-independent thylakoid membrane localization of transcripts encoding photosystem proteins (Mahbub et al., 2020), implying that they act cotranscriptionally on mRNA targeting before translation initiates. Likewise, Grad-seq experiments performed in *Salmonella* and *E. coli* showed that several RBPs reviewed here co-sediment with RNAP (Smirnov et al., 2016; Hör et al., 2020), which could indicate a cotranscriptional association of RBPs with nascent RNAs. Considering their transcript fate-determining activities, these RBPs emerge as potential candidates for promoting UTT.

#### Cold Shock Proteins

Cold Shock Proteins (CSPs) belong to an evolutionarily widespread family of small, acidic proteins that were originally discovered as mediators of cold shock response, but it is currently understood that their function exceeds adaptation to cold (reviewed in Ermolenko and Makhatadze, 2002; Horn et al., 2007; Budkina et al., 2020). Notably, the presence of at least one *csp* gene in the cell is essential for viability in *B. subtilis* (Graumann et al., 1997). The *E. coli* genome encodes nine different CSP genes, from which only four, CspA, CspB, CspG, and CspI, are induced by cold shock (Etchegaray and Inouye, 1999; Wang et al., 1999), and two, CspC and CspE, are constitutively expressed at 37°C (Yamanaka et al., 1994; Bae et al., 1999). Of note, the cold shock domain (CSD) in CSPs, confers a capacity to bind single-stranded nucleic acids (Jiang et al., 1997; Lopez et al., 1999; Phadtare and Inouye, 1999) and melt secondary structures within them (Phadtare et al., 2002a; Phadtare and Severinov, 2005; Rennella et al., 2017). Hence, in addition to mediating UTT as described below, CSPs may aid CTT by ensuring the transfer of unfolded mRNA from the nucleoid to ribosomes (El-Sharoud and Graumann, 2007).

Bioinformatical explorations unraveled a sequence-level bias towards U-richness in transcripts encoding integral membrane proteins in *E. coli* (Prilusky and Bibi, 2009) and *Lactococcus lactis* (van Gijtenbeek et al., 2016). Information published by our lab confirmed that the U-richness in membrane-traversing domains is important for their membrane localization, as predicted bioinformatically (Kannaiah et al., 2019). Interestingly, *E. coli* CspC and CspE preferentially bind U-rich artificial transcripts and endogenous membrane mRNAs (Benhalevy et al., 2015). Moreover, CspE overexpression causes the accumulation of ribosome-free transcripts encoding membrane proteins in the cytoplasm and positively affects their translation in the membrane (Benhalevy et al., 2017). This suggests that, *via* CspE mediation, these transcripts avoid CTT until they reach the membrane, where they are locally translated.

Current evidence suggests that CSPs promotes UTT by protecting nascent transcripts from RNases. For instance, overexpression of CspC and CspE increases the stability of transcripts encoding stress response proteins RpoS and UspA (Phadtare and Inouye, 2001). Whereas CspE levels remain constant through different growth phases, CspC levels increase upon entry into stationary phase, which results in the stabilization of *rpoS* transcripts (Shenhar et al., 2012). These transcript-stabilizing capabilities can be explained by the tendency of CspE to bind poly(A) sequences, counteracting the ribonucleolytic activity of PNPase and RNase E (Feng et al., 2001). Considering that most transcripts undergo polyadenylation (Mohanty and Kushner, 2006), CspE could act as a global molecular shield against the concerted poly(A)-dependent exoribonucleolytic action of PNPase in the 3'-end and the endonucleolytic attack of RNase E in internal cleavage sites of nascent transcripts. Of note, *cspE* mRNA is downregulated in the absence of PNPase (Polissi et al., 2003), suggesting that a regulatory mechanism balances the ribonucleolytic activity of PNPase and the anti-RNase protection by CspE.

For these activities to promote UTT, CSPs should access nascent transcripts as soon as they emerge from RNAP. Indeed, CspE binds nascent RNAs and acts as an antiterminator by melting secondary structures of intrinsic terminators (Hanna and Liu, 1998; Bae et al., 2000; Phadtare et al., 2002a,b; Phadtare and Severinov, 2005). Collectively, these pieces of evidence indicate that CSPs can promote the uncoupling of transcription and translation by counteracting RNase activity over transcripts and promoting translation-independent transcription over intrinsic terminators.

Cold shock proteins show UTT-promoting activities in other species. In *Salmonella*, CspC and CspE bind about 20% of the transcriptome with important regulatory implications for its pathogenicity (Michaux et al., 2017). For example, the *ecnB* mRNA, which is bound by these CSPs, shows lower transcript levels and stability in a *ΔcspCE* background. Levels and stability of this transcript were restored in *ΔcspCE* cells expressing a temperature-sensitive RNase E mutant at the restrictive temperature. Thus, CSPs protect *ecnB* transcripts from RNase E-mediated decay (Michaux et al., 2017). Similarly, CspE binds and stabilizes *yciF* transcripts, conferring *Salmonella* increased resistance to bile salts by impermeabilizing the cell membrane (Ray et al., 2019b). Also, in *B. subtilis*, CspB shows extranucleoidal localization in a transcription-dependent manner (Mascarenhas et al., 2001; Weber et al., 2001), suggesting that CspB binds transcripts upon entry into the cytoplasm.

All in all, CSPs have emerged as RBPs with important roles in gene regulation and, potentially, promoting UTT. Their function resembles that of FRGY2, a CSD-containing protein that binds mRNAs in the nucleous of frog oocytes and protects transcripts from degradation and translation in the cytoplasm until they are released in a regulated manner during oocyte development (Bouvet and Wolffe, 1994). Their multifaceted functions as transcription antiterminators and antiribonucleolytic shields make CSPs promising subjects for future study regarding their putative role as UTT facilitators.

#### Ribosomal Proteins

In addition to multiple interactions with rRNAs within the ribosome, ribosomal proteins (RPs) perform extraribosomal moonlighting functions as free RBPs, often involved in gene regulation (reviewed in Bhavsar et al., 2010; Aseev and Boni, 2011). Several RPs bind their cognate mRNAs to negatively autoregulate their translation and to keep RP homeostasis. In other cases, RPs play functions that could enable UTT.

One example is the S1 RP in *E. coli*, which binds single-stranded AU-rich sequences located upstream of SD sequences within the 5'-UTRs (Boni et al., 1991; Mogridge and Greenblatt, 1998; Komarova et al., 2002), an activity shown to increase the stability of the *lacZ* mRNA (Komarova et al., 2005), most likely due to the overlap between S1 binding sites and RNase E cleavage sites (Kaberdin and Bläsi, 2006). In agreement with this hypothesis, S1 binds *cspE* and *rpsO* transcripts and protects them against RNase E attack (Delvillani et al., 2011). S1 was further shown to counteract PNPase-mediated mRNA decay (Briani et al., 2008). When overexpressed, S1 binds several mRNAs, including the *pnp* mRNA itself, and increases their stability against PNPase-mediated decay. In line with these results, depletion of S1 leads to the destabilization of these transcripts *in vivo* (Briani et al., 2008). Similar to CspE, S1 binds poly(A) sequences (Kalapos et al., 1997), but this binding does not protect transcripts against PNPase degradation *in vitro* (Feng et al., 2001). Whether S1 counteracts PNPase *in vivo* by promoting CspE-dependent transcript protection (see above) or through an alternative mechanism remains unknown. Additionally, S1 was shown to increase protein secretion that is mediated by the hemolysin signal peptide, and this was accompanied by the stabilization of the corresponding transcripts (Khosa et al., 2018). If S1 can promote UTT, these RNA-protecting activities should be implemented as soon as the nascent transcript emerges from RNAP. In this regard, S1 associates with RNAP and promotes its transcriptional processivity (Sukhodolets and Garges, 2003; Sukhodolets et al., 2006). Additionally, S1 binds RNAP indirectly forming a ribonucleoprotein (RNP) with IsrA sRNA (van Nues et al., 2015). Thus, it is plausible that S1 binds nascent transcripts cotranscriptionally and protects them from ribonucleolytic attack, thus favoring the occurrence of UTT.

Another example is the S4 RP, which negatively autoregulates its translation by binding its cognate cistron within the α-operon and creating a pseudoknot that leads to the entrapment of an inactive translation initiation complex (Spedding and Draper, 1993; Schlax et al., 2001). This autoregulation also represses the translation of several other RPs cotranscribed within the same operon. However, the regulation of the *rpoA* gene, which encodes the α-subunit of RNAP and is also cotranscribed within the α-operon, is not subjected to this negative regulation (Thomas et al., 1987). Although the mechanism of this exclusion remains unknown, it implies that the α-operon can be subjected to partial disruption of CTT; i.e., whereas the α-subunit of RNAP is cotranscriptionally translated, the genes encoding ribosomal proteins are cotranscribed but translationally repressed by S4. To exert such CTT disruption, S4 would need to act cotranscriptionally. Indeed, S4 can bind RNAP and antiterminate Rho-dependent terminators (Torres et al., 2001), thus protecting ribosome-free nascent transcripts from PTT. Therefore, S4 plays a double role in promoting UTT: disruption of the transcription-translation coupling in the α-operon and preventing Rho-mediated termination of TECs that are not physically coupled to a translating ribosome.

The L4 RP also shows multiple activities that could promote UTT. L4 binds the regulatory CTD of RNase E and inhibits its activity, leading to stabilization of a subset of stress-related transcripts crucial for cell survival (Singh et al., 2009). Alleviation of the ribonucleolytic pressure on these mRNAs could allow a less tight CTT regime for these transcripts. Among the L4-stabilized transcripts is that of the triptophanase (*tna*) operon, which is subjected to an additional layer of regulation by L4 that is degradosome-independent. Specifically, L4 overexpression increases the stability of the *tnaCAB* transcript but causes translational repression of the TnaA protein by binding to the spacer between *tnaC-tnaA* (Singh et al., 2020). This could lead to the partial disruption of CTT in this operon, i.e., *tnaC* can be expressed by CTT, whereas *tnaA* translation is repressed despite the increase in its mRNA levels. Interestingly, this translational repression of *tnaA* takes place at early stationary phase to prevent the degradation of tryptophan, which is required for long-term survival through deep stationary phase (Singh et al., 2020), highlighting that CTT disruptions can have important physiological implications. Importantly, L4 binds its cognate transcript to attenuate its transcription (Lindahl et al., 1983). Thus, L4 could act cotranscriptionally as an antitranslation agent also over other nascent transcripts. Further investigation of extraribosomal functions of RPs should lead to a better understanding of these activities that are potentially involved in UTT.

#### Hfq

Besides its highly studied role as an sRNA-mRNA matchmaker (see below), Hfq exerts post-transcriptional regulation in *E. coli* by directly binding to transcripts and affecting their fate (reviewed in Kavita et al., 2018). Hfq was shown to bind the 5'-UTRs of its cognate transcript (Večerek et al., 2005) and of *cirA* (Salvail et al., 2013) and *mutS* mRNAs (Chen and Gottesman, 2017), as well as the ribosome binding site (RBS) of the Tn10 transposase mRNA (Ellis et al., 2015). In all these cases Hfq binding leads to translational repression of the target transcripts. These activities can be explained by the observation that in enterohemorrhagic *E. coli*, Hfq shows a preference for binding ARN triplets located in the proximity of RBSs (Tree et al., 2014), which could preclude translation initiation. Likewise, translational repression by Hfq plays a central role in the catabolic repression of the opportunistic pathogen *Pseudomonas aeruginosa*. With the assistance of the catabolic repression control protein Crc, Hfq binds specific A-rich sequences in the proximity of translation initiation regions to suppress translation of catabolite repressed genes (Sonnleitner and Bläsi, 2014; Pei et al., 2019).

Hfq was also shown to preferentially bind intrinsic terminator sequences in *Salmonella* (Holmqvist et al., 2016). Correspondingly, Hfq binds PAP I and promotes the synthesis of poly(A) tails at intrinsic terminators in *E. coli* (Hajnsdorf and Régnier, 2000; Le Derout et al., 2003; Mohanty et al., 2004). Analogously to CspE (see above), Hfq binds to poly(A) sequences overlapping with RNase E cleavage sites and confers protection against exoribonucleolytic decay by PNPase and RNase II (Folichon et al., 2003; Moll et al., 2003; Zhang et al., 2003). Collectively, this evidence indicates that Hfq binds intrinsic termination sites in order to promote polyadenylation and, by binding to these poly(A) sequences, protects 3'-UTRs and upstream sequences from ribonucleolytic decay.

Importantly, Hfq interacts with RNAP to promote transcription (Sukhodolets and Garges, 2003). Furthermore, Hfq shares topological similarities with YaeO, the only so-far discovered protein that binds and inhibits Rho in *E. coli* (Pichoff et al., 1998; Gutiérrez et al., 2007) and *Vibrio cholerae* (Pal et al., 2019). Accordingly, Hfq suppresses Rho-dependent termination by simultaneously binding Rho and AU-rich sequences located upstream *rut* sites (Rabhi et al., 2011). Recently, it has been shown that Hfq pervasively binds nascent transcripts in *E. coli* (Persson et al., 2013; Sedlyarova et al., 2016) and *P. aeruginosa* (Kambara et al., 2018; Gebhardt et al., 2020). The helix-like localization of Hfq observed in *E. coli* under certain growth conditions (Taghbalout et al., 2014; Malabirade et al., 2017; Kannaiah et al., 2019) resembles the helical-ellipsoidal conformation of the nucleoid (Fisher et al., 2013), reinforcing the idea that Hfq could indirectly associate with the chromosome by binding nascent transcripts. Similar to S1, Hfq forms an RNP with IsrA sRNA that associates with RNAP (van Nues et al., 2015). Therefore, Hfq emerges as a potential UTT-promoting factor by antiterminating ribosome-free transcription that could otherwise be terminated by Rho. The fact that Hfq can interact with nascent transcripts implies that the antitranslation and antiribonuclease roles of Hfq described here could come into play cotranscriptionally to disrupt CTT and protect nascent mRNAs from RNases.

#### CsrA/RsmA

Although initially discovered as a regulator of glycogen biosynthesis upon entry into stationary phase (Romeo et al., 1993), CsrA has emerged as a global post-transcriptional regulator implicated in multiple cellular functions (reviewed in Vakulskas et al., 2015; Romeo and Babitzke, 2018). CsrA has been shown to bind hundreds of transcripts in *E. coli* (Edwards et al., 2011; Potts et al., 2017), *Campylobacter* (Dugar et al., 2016), *Salmonella* (Holmqvist et al., 2016), and *Legionella* (Sahr et al., 2017) affecting their post-transcriptional fate and potentially promoting UTT.

The main regulatory activity of CsrA is *via* translational repression (Dugar et al., 2016; Potts et al., 2017). For example, CsrA binds the SD sequence of the *hfq* mRNA and inhibits its translation (Baker et al., 2007). Similarly, CsrA binds at two sites in the 5'-UTR, including the SD sequence, of transcripts encoding the transcriptional regulator NhaR, which responses to high sodium concentrations and alkaline pH (Pannuri et al., 2012). Likewise, CsrA inhibits translation of the iron storage *dsp* mRNA by binding the 5'-UTR region of the transcript (Potts et al., 2017; Pourciau et al., 2019). In enteropathogenic *E. coli* (EPEC), CsrA represses translation of the virulence effector NleA (Katsowich et al., 2017). Upon contact with the host, EPEC injects effectors to the host cell through a type III secretion system with the assistance of the effector-bound chaperone CesT. After releasing the effector, free CesT binds and inhibits CsrA, leading to derepression of NleA and its subsequent translation and translocation to the host cell (Katsowich et al., 2017). The CsrA homolog RsmA binds the 5'-UTR of *psl* mRNA in charge of the biosynthesis of a structural polysaccharide in *P. aeruginosa* biofilms and inhibits its translation by refolding the transcript structure so that the SD sequence is not accessible to ribosomes (Irie et al., 2010). In all examples discussed here, translation inhibition was not accompanied by decreased transcript stability. Consequently, this can lead to the accumulation of ribosome-free transcripts in the cytoplasm, resembling the phenomenon observed upon CspE and S1 overexpression (see above). Interestingly, the CsrA/RsmA-regulated transcripts discussed here encode proteins responding to environmental stimuli. Thus, it is tempting to speculate that CsrA/RsmA promote UTT and the accumulation of untranslated pools of mRNAs whose expression can be rapidly derepressed upon environmental challenges without transcriptional delay.

Furthermore, CsrA can also act as an anti-RNase shield (Esquerré et al., 2016; Potts et al., 2017). For instance, CsrA was shown to be essential for *E. coli* motility by regulating the *fhl* operon, the master operon for flagellum biosynthesis, by increasing the stability of *fhlDC* transcripts (Wei et al., 2001), and this is achieved by protecting these mRNAs from RNase E cleavage (Yakhnin et al., 2013). Likewise, CsrA stabilizes the *Legionella* iron uptake regulator *fur* mRNA by binding a specific site in the proximity of a potential RNase E site (Sahr et al., 2017). CsrA and RsmA were also shown to increase the stability of the STM3611 transcript in *Salmonella* (Jonas et al., 2010) and of the *hrpG* transcript, the master regulator of T3SS genes in *Xanthomonas*, respectively (Andrade et al., 2014).

CsrA also displays transcription-related activities, e.g., binding to the *gap* operon transcript in *Legionella* cotranscriptionally to counteract Rho-dependent transcription termination (Sahr et al., 2017) and to RNAP as an RNP with IsrA sRNA in *E. coli* (van Nues et al., 2015). Furthermore, in *P. aeruginosa*, RsmA was shown to bind over 500 nascent transcripts cotranscriptionally (Gebhardt et al., 2020). Collectively, these publications indicate that CsrA/RsmA are major gene regulators that act over nascent transcripts. Thus, it is plausible that the antitranslation, anti-RNase, and antitermination activities of CsrA/RsmA favor the occurrence of UTT. Further investigating the cotranscriptional activities of CsrA/RsmA should shed light on the detailed involvement of CsrA/RsmA in promoting UTT.

#### ProQ

ProQ, a FinO-domain protein that acts as an sRNA-mRNA matchmaker (Smirnov et al., 2017; Westermann et al., 2019; Melamed et al., 2020), has recently emerged as an important RBP, which binds a substantial fraction of the *E. coli* and *Salmonella* transcriptomes (Smirnov et al., 2016; Holmqvist et al., 2018), suggesting that it may be involved in UTT implementation.

ProQ recognizes structural features present in sRNAs and 3'-UTRs of mRNAs and increases their stability upon binding (Smirnov et al., 2016; Holmqvist et al., 2018; Bauriedl et al., 2020; Stein et al., 2020). Specifically, ProQ was shown to stabilize *cspE* mRNA by binding its 3'-UTR and preventing RNase II-mediated exoribonucleolytic attack (Holmqvist et al., 2018). Stability of *cspC*, *cspD*, and *ompD* was also reduced in *ΔproQ* background (Holmqvist et al., 2018), but whether ProQ stabilizes these transcripts by antiribonuclease protection awaits experimental confirmation. Furthermore, about a third of ProQ binding events take place in sites overlapping with RNase E cleavage sites (Chao et al., 2017; Holmqvist et al., 2018). Collectively, these anti-RNase activities resemble those displayed by FinO, which recognizes and binds a similar structural feature of its only target FinP antisense RNA and exerts anti-RNase E protection (Jerome et al., 1999; Arthur et al., 2011). Importantly, ProQ associates with RNAP *via* an RNP formed with IsrA sRNA (van Nues et al., 2015). Thus, it is plausible that ProQ accesses and binds nascent transcripts cotranscriptionally, promoting UTT by protecting untranslated transcripts against ribonucleolytic decay.

#### Nus Factors

Nus factors regulate transcription elongation by affecting RNAP pausing and promoting transcription termination/antitermination (Santangelo and Artsimovitch, 2011; Sen et al., 2014). NusA is an essential component of the antitermination complex that, together with NusB, NusE, NusG, and several ribosomal proteins, enhances rRNA transcription rates (Squires and Zaporojets, 2000; Paul et al., 2004). Besides, NusA promotes RNAP pausing, which favors the formation, stability, and efficiency of intrinsic terminators (Farnham et al., 1982; Schmidt and Chamberlin, 1987; Toulokhonov et al., 2001). As discussed in section Coordination of CTT, NusA also mediates transcription termination by Rho (Epshtein et al., 2010; Hao et al., 2020; Said et al., 2020). Yet, NusA could possibly counteract Rho-dependent termination in certain instances. Specifically, NusA mutants with increased affinity for binding NusA utilization (*nut*) sites decreased Rho-dependent termination at specific cases where *nut* and *rut* sites overlap (Qayyum et al., 2016). Thus, regarding UTT, NusA could facilitate translation-independent transcription of mRNAs by interfering with certain Rho-dependent termination events on nascent ribosome-free transcripts.

Other Nus factors are involved in processive antitermination (PA) mechanisms. Opposite to dedicated antiterminators, PA factors associate with and modify TECs to promote transcriptional readthrough over multiple transcription terminators distally located in the operons under their regulation (Goodson and Winkler, 2018). For example, the NusG paralogue LoaP regulates transcription through termination sites located within two antibiotic biosynthesis operons in *Firmicutes*, *Actinobacteria*, and *Spirochaetes* (Goodson et al., 2017). Deletion of *loaP* led to a reduction in transcript levels of LoaP regulons. LoaP is thought to processively antiterminate intrinsic terminators, an activity that requires the 5' leader sequence of the transcripts under its regulation (Goodson et al., 2017). All in all, the PA activity of such Nus paralogs on their specific target operons could favor UTT by alleviating the need for a translationally coupled ribosome for counteracting intrinsic and Rho-dependent terminators.

### *Cis*-Acting RNA Elements

Riboswitches are regulatory elements located in the 5'-UTR of mRNAs that are comprised of two modules: a structurally complex aptamer that binds a ligand and an expression platform that is regulated by the aptamer structural folding. Ligand binding causes structural refolding of the aptamer, which affects the expression of the downstream expression platform in an ON/OFF manner (Sherwood and Henkin, 2016). Some riboswitches act by translationally repressing the ORF under their regulation (Breaker, 2018). For example, in cobalamin riboswitches, the SD sequence is sequestered within the aptamer structure (Johnson et al., 2012), which becomes accessible for ribosomes only upon ligand binding. In thiamin pyrophosphate riboswitches, on the other hand, an anti-SD sequence within the aptamer structure anneals to and folds over the SD sequence located in the expression platform and inhibits translation initiation (Winkler et al., 2002). Again, ligand binding causes the refolding of the aptamer and liberates the SD sequence for ribosome binding. Similar to riboswitches, RNA thermometers are 5'-UTR elements that undergo structural refolding and affect downstream gene expression, but their refolding is caused by changes in temperature rather than ligand binding (Kortmann and Narberhaus, 2012). For example, the transcription of the cold-induced *cspA* gene takes place at all temperatures, but the *cspA* transcript is highly unstable at 37°C due to RNase E-mediated decay (Fang et al., 1997). Upon cold shock, the *cspA* transcript undergoes substantial refolding and is stabilized in a more RNase-resistant folding that allows translation of the protein (Giuliodori et al., 2010). Oppositely, the translation of *rpoH* transcript, coding for the heat shock sigma factor, is repressed at physiological temperatures by a secondary structure that sequesters the SD sequence. Upon temperature upshift, this structure refolds in a way that exposes the SD sequence for translation initiation (Morita et al., 1999). Likewise, several virulence factors are regulated by RNA thermometers that enable translation at 37°C upon entry in warm-blooded mammalian hosts (Johansson, 2009). Importantly, riboswitch structures are folded cotranscriptionally (Mironov et al., 2002; Frieda and Block, 2012; Watters et al., 2016; Uhm et al., 2017; Ray et al., 2019a). Considering that RNAP pauses at SD sequences and start codons (Larson et al., 2014), it is likely that riboswitch regulation comes into action shortly after they emerge from RNAP and before the translation initiation. Hence, it is reasonable to argue that riboswitches and RNA thermometers can cotranscriptionally block translation and protect transcripts from ribonucleolytic attack, which would promote UTT. Such inert transcripts would be activated upon environmental changes that induce their translation.

Similarly, *cis*-acting RNA elements act as translational repressors of type I toxin-antitoxin (TA) systems (Masachis and Darfeuille, 2018). In many type I TA systems the primary toxin transcript is translationally inert, and this is achieved by sequestering the SD sequence in a secondary structure formed with an anti-SD sequence located upstream in the transcript (Gultyaev et al., 1997; Darfeuille et al., 2007; Shokeen et al., 2008; Kristiansen et al., 2016; Wen et al., 2017). In other cases, the translation of the toxin is repressed by structures formed by interactions between the 5'- and 3'-ends of the full-length mRNA (Thisted et al., 1995; Franch and Gerdes, 1996; Gultyaev et al., 1997). The high structural complexity of toxin mRNAs is also thought to prevent the interaction of the nascent toxin transcript with the template DNA, which avoids the formation of deleterious R-loops, and to confer increased antiribonuclease resistance (Masachis and Darfeuille, 2018). Thus, structural features of toxin mRNAs ensure their translationally-inert transcription and protection against RNases until downstream activation events trigger post-transcriptional translation of these transcripts.

Some *cis*-acting RNA elements could facilitate UTT in a more indirect manner. For example, inhibitory RNA aptamers (iRAPs) interact with RNAP and facilitate Rho-dependent termination (Sedlyarova et al., 2017). Interestingly, many iRAPs map to the antisense strand and curb antisense transcription, which reduces transcriptional interference and favors sense transcription (Sedlyarova et al., 2017; Magán et al., 2019). Considering translation-independent TECs are less likely to reinitiate transcription after clashing with an antisense TEC (Hoffmann et al., 2019), diminishing antisense transcription by antisense iRAPs can alleviate the requirement of a leading ribosome that supports the TEC conducting sense transcription.

Other *cis*-acting RNA elements not only have the potential to attenuate CTT but enforce its disruption. For instance, the *Salmonella* virulence *mgtCBR* operon harbors a leader region that acts as a Rho-antagonizing RNA element (RARE) and a *rut* site that is necessary for Rho-mediated transcription termination of this operon (Sevostyanova and Groisman, 2015). The RARE counteracts termination by trapping Rho in a termination-defective conformation. Importantly, translation of the *mgtCBR* transcript sequesters the RARE in a stem-loop (Sevostyanova and Groisman, 2015). Thus, the only manner to express this operon is by UTT, in a way that the RARE inhibits Rho-mediated termination and allows translation-uncoupled transcription of the full mRNA, which would be translated post-transcriptionally. The *corA* operon of *Salmonella* is subjected to a very similar regulation and its leader region is highly conserved in enterobacteria (Kriner and Groisman, 2015), so such strictly UTT-dependent gene expression could be relatively widespread in these species.Lastly, besides elements that act against individual termination sites, other *cis*-acting RNA sequences are involved in PA (see above Goodson and Winkler, 2018). For example, in *B. subtilis* the *eps* operon, encoding exopolysaccharide biosynthesis proteins, is regulated by an *eps*-associated RNA (EAR) sequence, located in the intergenic region between the second and third genes of the operon (Irnov and Winkler, 2010). The EAR sequence is necessary for transcription of the entire operon, as it antiterminates several intrinsic terminators located in distal sites within the operon. The EAR sequence is also able to antiterminate heterologous terminators, supporting the hypothesis that it acts as a PA (Irnov and Winkler, 2010). Such *cis*-acting RNA elements with PA activities could promote UTT in the operons that they regulate since they would alleviate the requirement of a cotranscriptionally coupled ribosome to counteract terminators.

### *Trans*-Acting RNA Elements

sRNAs are the archetypical example of *trans*-acting RNAs in bacterial gene regulation. They are typically 50–300 nt long transcripts that determine transcript fate by imperfect base-pairing with target mRNAs (Wagner and Romby, 2015). This process is often facilitated by the mRNA-sRNA matchmakers Hfq (Updegrove et al., 2016) and ProQ (Holmqvist et al., 2020). More recently, matchmaking activity has been reported for CsrA (Müller et al., 2019). sRNAs can affect the stability and/or translation of target mRNAs either positively or negatively, and these activities can potentially promote UTT.

In *Salmonella*, the glucose-responsive sRNA SgrS stabilizes the *pdlB-yigL* bicistronic transcript by preventing RNase E-mediated cleavage (Papenfort et al., 2013). Similarly, the sRNA RydC basepairs with the 5'-UTR of the *cfa* mRNA and stabilizes the longer isoform of this transcript by counteracting RNase E attack (Fröhlich et al., 2013). In *B. subtilis*, the sRNA RoxS binds the 5'-end of the *yflS* mRNA and prevents exoribonucleolytic decay by RNase J1 (Durand et al., 2017). Besides anti-RNase protection, the *B. subtilis* sRNA SR1 blocks translation binds in the 5'-UTR of its target *ahrC* mRNA and blocks its translation (Heidrich et al., 2006, 2007). Likewise, in *Legionella pneumophila*, the competence operon is translationally repressed by the sRNA RocR (Attaiech et al., 2016). sRNAs can further promote translational repression by recruiting Hfq to binding sites that prevent ribosomes association with SD sequences of their mRNA targets (Desnoyers and Massé, 2012; Azam and Vanderpool, 2018).

As sRNAs are relatively short and not translated by ribosomes they can penetrate the nucleoid mesh (Sheng et al., 2017) and putatively engage in cotranscriptional processes. Indeed, it was recently discovered that the sRNAs DsrA, ArcZ, and RprA, although induced by different stresses, bind the 5'-UTR of nascent *rpoS* transcripts to suppress Rho-dependent termination and allow expression of the stationary phase sigma factor (Sedlyarova et al., 2016). The expression of Rho itself is subjected to similar regulation, as the sRNA SraL basepairs with the 5'-UTR of the *rho* transcript to antiterminate its transcription (Silva et al., 2019). Thus, sRNAs could promote UTT by counteracting pervasive Rho-mediated transcription antitermination of ribosome-free mRNAs. The cotranscriptional association of sRNAs with mRNAs also suggests that they could exert their antitranslation and antiribonucleolytic regulation over nascent transcripts, facilitating the occurrence of UTT.

Interestingly, the *E. coli* sRNA IsrA associates with RNAP and forms RNPs together with important transcript fate-determining proteins Hfq, S1, CsrA, ProQ, and PNPase (van Nues et al., 2015). The association of these RNPs with RNAP and their corresponding regulatory outputs await further characterization. Yet, besides directly acting as UTT-inducing factors, sRNAs could promote UTT by serving as landing and integration platforms to enable cotranscriptional action of proteins with antitranslation, anti-RNase, and antitermination functions.

### Bacterial RNP Bodies

Biomolecular condensates formed by multivalent interactions among proteins and nucleic acids have recently gained special interest. These condensates assemble and dissolve according to LLPS principles and they are involved in important cellular functions, including RNA metabolism (Banani et al., 2017). For example, processing bodies (P-bodies) and stress granules have been shown to sequester poorly translated transcripts for decay or storage in eukaryotes (Decker and Parker, 2012; Khong and Parker, 2020).

Biomolecular condensates show selective permeability and concentrate biomolecules and processes to discreet subcellular regions (Banani et al., 2017). Thus, they are attractive tools for prokaryotes to gain spatial complexity in their cytoplasms, which are generally devoid of membrane-bound organelles. Indeed, the existence of Bacterial RNP bodies (BR-bodies) in bacteria has been recently reported (Al-Husini et al., 2018; Muthunayake et al., 2020). In *C. crescentus*, RNase E assembles into BR-bodies together with the degradosome components and poorly translated RNAs, creating P-body-like condensates engaged in RNA degradation (Al-Husini et al., 2018, 2020). These RNase E condensates associate with the *C. crescentus* nucleoid in the proximity of rDNA loci (Bayas et al., 2018). Interestingly, the *E. coli* RNase E, which unlike in *C. crescentus* localizes to the inner membrane together with the other degradosome components, forms clusters that show RNA-dependent assembly and dynamics (Strahl et al., 2015), resembling the *C. crescentus* BR-bodies, thus suggesting that *E. coli* degradosomes may form condensates by LLPS in the inner membrane. Endoribonucleolytic attack by RNase E is believed to be the initial and the rate-limiting step of RNA degradation, so it is reasonable to argue that proximity to these RNase E condensates may increase the likelihood of a transcript to undergo decay. In agreement with this, *E. coli* membrane-localizing transcripts show a lower average stability, and artificially targeting cytoplasmic mRNAs to the membrane increases their degradation rate (Moffitt et al., 2016). Additionally, detaching RNase E from the membrane sensitized cytoplasmic ribosome-free transcripts (Hadjeras et al., 2019). Collectively, this evidence indicates that RNase activity is highly localized within bacterial cells by the mediation of BR-bodies, and that regions distant to RNA-degrading condensates may not be subjected to intensive ribonucleolytic pressure. This would certainly support UTT, especially in those organisms where the core ribonucleolytic machinery localizes in the membrane, as nascent ribosome-free transcripts would not be reachable for degradosomes and, hence, substantial antiribonucleolytic protection would not be required.

Furthermore, the bacterial cytoplasm shows glass-like properties and its fluidity varies according to the physiological state of the cell, where higher metabolic activity correlates with higher fluidity of the cytoplasm and vice versa (Parry et al., 2014). These biophysical properties may affect the function of biomolecular condensates, i.e., more fluid, liquid-like condensates allow higher motion and enzymatic activities, whereas more solid, aggregate-like entities specialize in storage (Banani et al., 2017). Bacteria undergo a notable metabolic slowdown upon entry into stationary phase or stress. This leads to the glassification of the cytoplasm (Parry et al., 2014), which could convert BR-bodies into RNA storage condensates rather than RNA processing bodies (Muthunayake et al., 2020). Such RNA-storing BR-bodies could further support UTT by accumulating and protecting untranslated transcripts until favorable conditions permit translation reinitiation.

## Final Remarks

Although the individual processes leading to gene expression are subjected to extensive study, less is known about how these processes affect each other and how living cells spatiotemporally arrange the machineries that execute these functions. Yet, it is now acknowledged that these processes regulate each other and that their correct interplay is crucial for cell viability (Dahan et al., 2011). In this regard, prokaryotic CTT remains a paradigmatic example of such crosstalk. A proper CTT regime is of crucial importance for the overall cellular function, and is maintained over different growth conditions. Thus, upon environmental challenges that independently affect transcription or translation, bacteria quickly coordinate the kinetics of the unaffected process accordingly (see section Coordination of CTT). The radically different CTT regimes showed by different organisms (Johnson et al., 2020; O’Reilly et al., 2020; Wang et al., 2020b; Webster et al., 2020) reflect the underlying diversity in gene expression regulatory mechanisms among different species. Hence, further studying the physiological and molecular factors that mediate and regulate CTT is paramount. Of note, a transcriptome-wide assessment of the actual occurrence of CTT remains pending.

As a prokaryotic-specific phenomenon, CTT arises as an interesting target for the development of antimicrobial therapeutics and, for instance, targeting (p)ppGpp-mediated CTT regulation shows great potential. Alarmone-deficient strains are metabolically compromised but viable, so, unlike compounds that target essential targets, targeting (p)ppGpp metabolism may greatly reduce cell viability without exerting the selective pressure that leads to the arousal of antibiotic resistance (Hauryliuk et al., 2015; Syal et al., 2017).

Then again, studying evolutionarily shaped mechanisms that promote UTT (section Mechanisms Potentially Enabling UTT) can offer hints of how to purposely disrupt CTT under conditions that it is essential. Most likely, these mechanisms act in cooperation. In some cases, simultaneous action of UTT-promoting factors may be required, as suggested by the existence of RNA-mediated Hfq-CsrA complexes in *E. coli* (Caillet et al., 2019) and the partial overlap of Hfq and RsmA targetomes in *P. aeruginosa* (Gebhardt et al., 2020). Likewise, overexpression of S1 leads to the accumulation of ribosome-free *cspE* transcripts with increased stability, indicating that this population is subjected to simultaneous anti-translation and anti-RNase protection (Delvillani et al., 2011). RNPs that associate with RNAP, such as those mediated by IsrA sRNA (van Nues et al., 2015), could provide proper ground for such cooperation. Alternatively, UTT could be the result of tandem action of these factors, as in the case of the *L. pneumophila* RocC, a FinO-domain protein that protects the RocR sRNA from ribonucleolytic degradation, so that RocR subsequently represses translation of the competence operon (Attaiech et al., 2016). Similarly, *cspE* and *cspC* mRNAs are among the transcripts stabilized by the L4-mediated inhibition of RNase E (Singh et al., 2009), and *cspE* transcripts are also stabilized by S1 (Briani et al., 2008). Besides, ProQ also protects *csp* transcripts from RNases (Holmqvist et al., 2018). Thus, RPs and ProQ appear to be upstream activators of CSP-mediated UTT.

By now, the notion that prokaryotes have intricate intracellular organization is well-acknowledged (Rudner and Losick, 2010; Govindarajan and Amster-Choder, 2016; Surovtsev and Jacobs-Wagner, 2018). Consequently, prokaryotic UTT implies that the spatiotemporal separation between transcription and translation emerged earlier than the arousal of the eukaryotic cell. This is supported by the partial conservation of Rbp2 and Rbp3, RBPs mediating translation-independent RNA localization in cyanobacteria (Mahbub et al., 2020), in the eukaryotic algae *Chlamydomonas*, where they also bind and localize mRNAs to specific sites where local translation takes place (Uniacke and Zerges, 2009). Hence, it is likely that certain RNA-localizing pathways operating in eukaryotes originated from prokaryotic CTT-disrupting mechanisms. Further investigating these events in prokaryotes may unmask novel mechanisms operating at a suborganellar scale in eukaryotes.

In the past, researchers have drawn a clear line between transcriptional and post-transcriptional regulatory mechanisms in bacteria. In the light of the emerging evidence, we posit that many post-transcriptional regulatory mechanisms come into action on nascent transcripts and that their cotranscriptionality has so far been ignored. As already recognized in eukaryotes (Choder, 2011), factors acting cotranscriptionally can determine transcript fate in prokaryotes as well. Half a century after Miller’s famous micrographs (Miller et al., 1970), we foresee that the study of prokaryotic CTT and UTT will doubtlessly produce novel insights that will reshape our understanding of prokaryotic gene expression and subcellular organization.

## Author Contributions

MIO and OAC wrote the original draft and edited the manuscript. OAC coordinated the work and acquired funding. All authors contributed to the article and approved the submitted version.

### Conflict of Interest

The authors declare that the research was conducted in the absence of any commercial or financial relationships that could be construed as a potential conflict of interest.
